# Biomechanical analysis of skull trauma and opportunity in neuroradiology interpretation to explain the post-concussion syndrome: literature review and case studies presentation

**DOI:** 10.1186/s41747-020-00194-x

**Published:** 2020-12-08

**Authors:** Yannick Distriquin, Jean-Marc Vital, Bruno Ella

**Affiliations:** 1 Laboratory of Anatomy, School of surgery, Bordeaux University, 146 rue Léo-Saignat, 33076 Bordeaux Cedex, France; 2 Department Head of Spinal Pathology and Spine Surgery, University Hospital Center of Bordeaux, Place Amélie Raba Léon, 33076 Bordeaux Cedex, France; 3 Department Head of Oral Medicine and Surgery, University Hospital Center of Bordeaux, 1, rue Jean Burguet, 33075 Bordeaux Cedex, France

**Keywords:** Craniocerebral trauma, Finite element analysis, Post-concussion syndrome, Skull, Tomography (X-ray, computed)

## Abstract

Traumatic head injuries are one of the leading causes of emergency worldwide due to their frequency and associated morbidity. The circumstances of their onset are often sports activities or road accidents. Numerous studies analysed post-concussion syndrome from a psychiatric and metabolic point of view after a mild head trauma. The aim was to help understand how the skull can suffer a mechanical deformation during a mild cranial trauma, and if it can explain the occurrence of some post-concussion symptoms. A multi-step electronic search was performed, using the following keywords: biomechanics properties of the skull, three-dimensional computed tomography of head injuries, statistics on skull injuries, and normative studies of the skull base. We analysed studies related to the observation of the skull after mild head trauma. The analysis of 23 studies showed that the cranial sutures could be deformed even during a mild head trauma. The skull base is a major site of bone shuffle. Three-dimensional computed tomography can help to understand some post-concussion symptoms. Four case studies showed stenosis of jugular foramen and petrous bone asymmetries who can correlate with concussion symptomatology. In conclusion, the skull is a heterogeneous structure that can be deformed even during a mild head trauma.

## Key points

- Biomechanical resistance properties of cranial bones and sutures are heterogeneous.
- The skull base could be the site of a major bone shuffle after a head trauma.
- Three-dimensional computed tomography of the skull base in four case studies showed foramen stenosis and asymmetry, explaining some post-concussion syndrome symptoms.

## Background

Dewan et al. [1] reported that 68 million individuals worldwide are estimated to sustain traumatic brain injuries (TBI) from all causes each year. Mild TBI (MTBI) is the most common, with a Glasgow Coma Score ranging from 13 to 15. This means that neurovascular lesions are partial or absent despite there having been a transient loss of consciousness. The follow-up of patients and athletes having suffered these injuries reveals a major medical and social impact for these victims [2, 3]. The sequelae of these injuries are defined as post-concussion syndrome (PCS), which is characterised by the persistence of symptoms several months after the trauma. These disturbances can affect numerous neurological functions, leading to significant social and family problems [4, 5]. Currently, no or few anatomical and clinical lesions are identified when additional investigations are performed. Recent improvements show that vascular disturbances and brain axonal architecture seem to be an explanation for the post-traumatic chronic symptoms [6].

Today the diagnostic difficulties result in poor medicolegal recognition of the syndrome, which is essentially psychopathological [7]. The aim of this literature review is to propose a mechanical understanding of bone and suture disruptions during MTBI. The analysis of published biomechanical, radiological, statistical and normative studies allowed us to focus on the changes and constraints to which the base of the skull is subjected. The results encouraged us to formulate the hypothesis that a mild head trauma can induce jugular foramen stenosis and petrous bone asymmetries, which can explain some symptoms of PCS.

## Methods

### Literature search

In order to perform a narrative review of available literature on head trauma, we have selected articles through a multistep process. The first author had a web-based literature database that has been continuously updated from 2016 to late 2019. Articles included were searched from PubMed/Medline, Scopus, Cochrane Database, Google Scholar, and ResearchGate database according to the subject heading. Initially, the search field focused on computer analyses examining the resistance of the skull. The key words were ‘biomechanical property of skull’. Then we searched for radiological studies concerning head injuries using the key words ‘radiology of skull fracture’. We completed our research with the key words ‘statistical analysis of skull fracture’ to locate the most impacted region of the skull in head trauma. Finally, we included normative analyses of the skull base, using the key words ‘normative measurement of skull base’.

### Eligibility criteria

We used predefined criteria to determine article eligibility. Following a review of their title and abstracts, we excluded studies older than 20 years of age, those performed on animals and those related to craniosynostosis and cranial surgery. Secondly, after reading the full text, we selected the studies which presented the most interesting data, results, illustrations and conclusions. It resulted in a qualitative and subjective choice of studies, including those with well-elaborated and interesting learning points.

### Data extraction and analysis

The eligible studies and corresponding data were extracted by two authors. The choice of studies included was done by the first author. For a clearer presentation, we subdivided the results of the review review into four sections: biomechanical, radiological, statistical and normative studies.

### Case studies

In addition, we propose an analysis of four case studies of three-dimensional (3D) computed tomography (CT) of the skull base after minor head trauma. All these four patients were suffering from a PCS. We studied the symmetry of the skull base, particularly the temporal bone and jugular foramen size after head trauma, which can increase post-concussion symptoms. We have tried to establish the link between local impact, skull base deformations and post-concussion symptoms described by the patients. In the illustrations, we have represented the impact localisation and we have measured the angle between temporal petrous bone and the middle axis of the skull base. For the tension headache, we have compared the perimeter between both jugular foramina. The tomography has been converted and extracted with Horos 2K v2.1.1. software [The HorosProject].

## Results

### Literature search and assessment

**Fig. 1:**
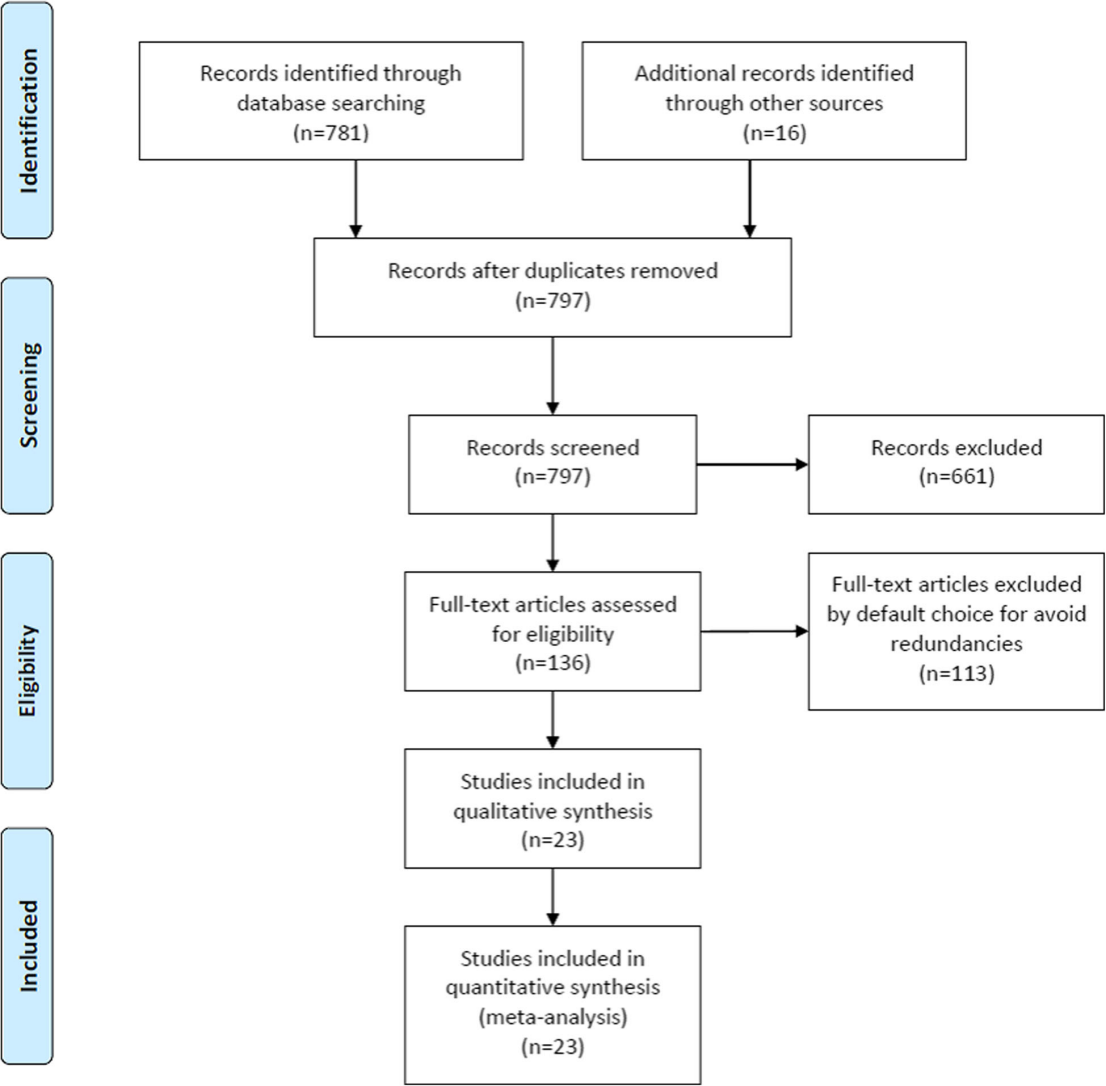
Research and reference classification using the PRISMA model

Literature search in 797 articles using the key words. After inclusion and exclusion criteria were applied, 661 publications were excluded based on their titles and abstracts. A total of 136 eligible articles were found and a complete analysis allowed us to achieve a qualitative choice. Twenty-three articles [8–30] were selected and displayed (Fig. 1, Tables 1 and 2).

**Table 1:** Qualitative inclusion criteria

First author, year of publication	Variability of bone resistance	Bone resistance > suture resistance	Resistance according to the morphology of the skull	Resistance of skull base < resistance of calvaria	Deformability of skull	Normative study of skull base	Skull base deformations could bring post-concussion symptoms
Lillie et al. 2016 [16]	+					+	
Wanyura et al. 2012 [9]			+	+	+		
Coats and Margulies 2006 [10]	+	+			+		+
Davis et al. 2012 [11]	+	+			+		
Delye et al. 2015 [12]	+	+			+	+	
Song et al. 2015 [13]		+			+		
Zhang 2015 [14]		+	+	+	+		
Sahoo et al. 2015 [15]	+			+	+		
Mullroy, 2012 [16]		+			+		
Orman et al. 2015 [17]		+			+		
Idriz et al. 2015 [18]		+	+	+	+	+	+
Simon, 2018 [19]	+			+			
Leibu et al. 2017 [20]	+			+			
Sim et al. 2017 [21]	+	+		+			
Meyer et al. 2019 [22]						+	
Russo and Smith 2011 [23]						+	
Skratz, 2016 [24]						+	
Gupta et al. 2014 [25]						+	
Papini et al. 2017 [26]						+	+
Sepadari, 2013 [27]						+	+
Edwards et al. 2018 [28, 29]						+	+
Coello et al. 2010 [30]	+				+		+

**Table 2:**
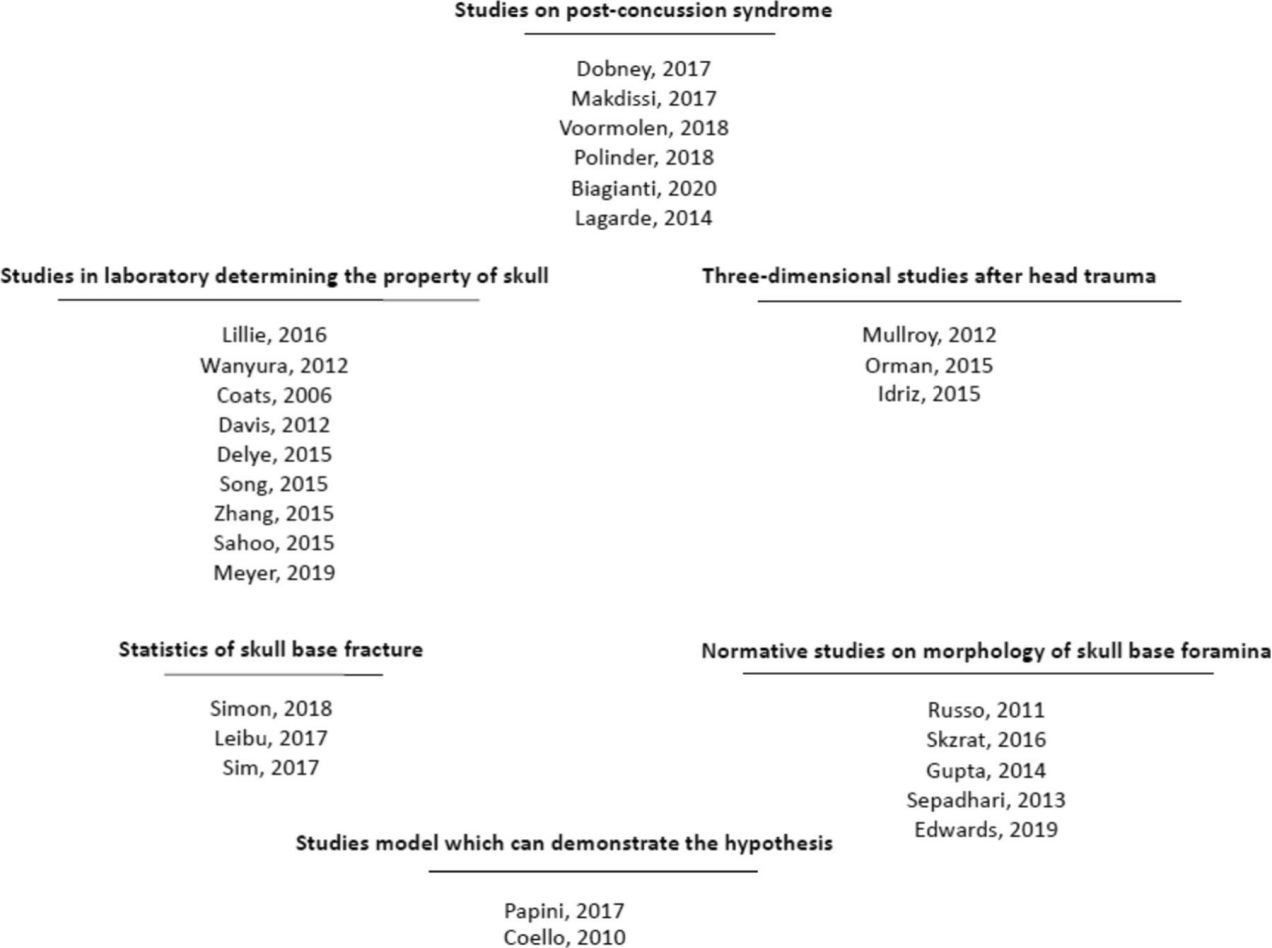
Results of search strategy using the PICO model

PICO: P = patient (or population, or problem); I = intervention (or prognostic factor, or exposure; C = comparison; O = outcome (https://lhncbc.nlm.nih.gov/publication/web-interface-pico-patient-intervention-comparison-and-outcome)

### Study characteristics

Nine biomechanical studies [8–15, 22] have allowed us to understand the heterogeneous architecture of the skull, its ossification [8], shape [9], sutures [10–14], foramen [9, 15] and its cervical spinal supports [22]. Three 3D CT studies [16–18] showed diastatic fractures. Three statistical studies [19–21] showed that the petrous part of the temporal bone is frequently impacted by cranial trauma. Six normative studies (Ref [23–29]) showed that there are some standard values about the size of the skull base foramens. Two studies [26, 30] were using a process that can be utilised to explain some PCSs.

### Biomechanical studies

The studies concerning the resistance of the skull have provided numerous data about the evolving properties of its bones and sutures. The first scientific studies presented concern the evolution of skull ossification depending on age and gender. Lillie et al. [8] have described ossification variations based on gender, age, and musculoskeletal stresses. The objective of this study was to integrate variations in bone resistance in computer models. The measurements performed indicate that the skull bones thicken primarily in regions subject to the greatest musculoskeletal stresses. The base of the skull undergoes the greatest ossification under the effect of ascending spinal pressures and descending musculofascial tractions. Using a finite element model, Wanyura et al. [9] have analysed the absorption and distribution of stresses during an impact causing a cranio-orbital fracture. The skull model presents a homogeneous and isotropic resistance. It incorporates the principal geometric bone variations (volume, thicknesses, main foramen) without taking into account suture notions. The results indicate very high-stress concentrations on the frontal bone (point of impact), the ethmoid bone, and the jugular foramen. Due to its form, the bone structure of the skull is subject to stresses remote from the point of impact. The presence of many foramina (foramen magnum, two jugular foramina, and two foramen lacerum) at the centre of the skull base may explain why a part of traumatic stress is concentrated in the skull base.

In paediatrics, the notion of suture resistance depends on the suture ossification that occurs during growth, up to the age of 25 years for certain sutures. Coats et al. [10] determined several comparisons between bone and suture resistances. Paediatric cranial bone resulted to be 35 times stiffer than paediatric cranial suture [10]. This result led the authors to conclude that paediatric cranial sutures distort 30 times more before the paediatric cortical bone fractures. In addition, in adults, the bones of the calvaria have resistance properties similar to those of the sutures. This means that there is a significantly reduced risk of deformation of the cranial vault.

More recently, Davis et al. [11] measured the flexural resistance and elastic modulus of the cortical bones (single and triple-layer) and sutures in a 6-year-old child. The results reveal significant differences in rigidity and elasticity between the sutures and the bone. These findings suggest that the sutures of a 6-year-old skull are the regions most susceptible to fracture and that is due to their tendency to remain connected beyond the point of failure [11]. The authors of these two aforementioned studies therefore concluded that, in paediatrics, diastatic fractures are likely to be more frequent but much more difficult to diagnose by two-dimensional imaging.

Delye et al. [12] created a normative database concerning bone and cranial suture density from age 0 to 20 years. This process enables the variation in mechanical resistance between the bone and the suture to be defined. The same process could be extended to adult suture resistance in order to define a risk scale by age of life. Song et al. [13] measured the influence of the sinuses during maxillary, zygomatic and frontal traumas. A finite element model experiment showed a diagram measuring the displacement of the bones in centimeter, as a function of the force exerted. The skull is subject to the same laws of physics as other materials: the suture has elastic resistance enabling interosseous displacement up to the elasticity limit (3 cm for 4 × 10^5^ Newton) [13]. The study helps us understanding that sutures resist by bending, before failing if the force applied ultimately reaches its maximum elasticity limit. Beyond this limit, the suture breaks and causes a diastatic fracture.

Zhang et al. [14] analysed the distribution of mechanical stresses according to suture geometry. In this study, differences in suture resistance to traction/compression loading have been calculated by a finite element model as a function of their morphology (sinusoidal or straight). These morphologies were studied because the sutures of the cranial vault are sinusoidal, whereas the sutures of the skull base are straight. This study reveals that sutures with interdigitations are more resistant than straight sutures when traction and compression forces are applied. In this study, shear stresses were not studied, but it can be imagined that there are significant disparities in resistance due to the presence of interdigitations. Notably, during a traumatic injury, the forces are often perpendicular. The sutures of the calvaria are at the angle, whereas the basilar sutures are more vertical. Hence, the cranial vault is likely to absorb the impact by displacement with the subduction of the bone plates, whereas the base of the skull may distort by vertical and horizontal slipping. As a consequence, it can be hypothesised that the skull base sutures may be more deformable than the cranial vault sutures.

Sahoo et al. [15] have provided results that further improve our understanding. This study assesses the properties of the skull during a lateral impact depending on the population concerned and the type of trauma (force and shape of objects). The tests have been performed on a model based on the biomechanical references of 17 embalmed skulls. The results reveal two major stress concentrations around the temporal bone. They are located in front of and behind its petrous part, at the level of its occipital and sphenoidal sutures. A synopsis of the various biomechanical studies on the skull indicates us that the base of the skull is a region liable to be subject to significant stresses, while at the same time possessing a greater deformation capacity than the cranial vault.

### Radiological studies

Mulroy et al. [16] and Orman et al. [17] analysed and compared 3D and 2D CT reconstructions of children having suffered a head injury. Both studies conclude that diastatic fractures may be underdiagnosed, consistent with the intuition of Coats et al. [10] and Davis et al. [11]. In the article by Mulroy et al. [16], a 3D CT reconstruction of a 22-month-old child having fallen from a height of 15 cm onto the vertex is shown. The image presented shows three peri-parietal diastatic fractures. In this case, a fall of 15 cm could easily have been considered as a mild injury with conventional radiological interpretations. The study conducted by Idriz et al. [18] describes the various paediatric cranial sutures using 3D and 2D CT reconstructions. The authors describe the anatomical specificities of each paediatric cranial suture. The relevant anatomical variations of the skull base during growth lead the authors to emphasise the importance of comparing the symmetry and the knowledge of the anatomic appearances of the basal suture for avoiding misdiagnosis [18].

### Statistical studies

Statistical studies confirmed that the base of the skull may undergo significant deformation. Simon et al. [19] analysed basilar skull fractures using epidemiological statistics. Basilar skull fractures are present in 19–21% of cases, despite the fact that this region has infrequent exposure to direct trauma. However, they only concern 4% of serious injuries, suggesting that this region may better absorb the kinetic energy of the trauma with minimum lesions. Leibu et al. [20] analysed 196 skull base fractures. with 116 fractures located in the middle temporal fossa. In the large majority of cases (60%), the cranial structure is subject to significant stresses around the petrous part of the temporal bone. Sim et al. [21] confirmed these results via an analysis of 292 cranial fractures in children under the age of 12 years. The analysis and observation of diastatic fractures reveal that the occipitomastoid suture is the most commonly concerned (45%), followed by the lambdoid suture (35%). Simon et al. [19] specified that 50% of skull base fractures are associated with another central nervous system injury, and about 10% have cervical spine fractures. Meyer et al. [22] measured stresses on the cervical spine during an impact using a finite element model including the weight of the skull. The authors showed that the suboccipital joints undergo significant shear and flexural stresses, recalling the idea of the pressure forces between the cervical spine and the base of the skull. In a head trauma, if the cervical spine is damaged, this means that the base of the skull has transmitted traumatic stress to the spine. The authors concluded highlighting the importance of the shear forces exerted on the suboccipital and basilar region.

### Normative studies

Perfect bilateral symmetry in the body is basically an only theoretical concept, which practically does not exist in live organisms. Knowledge of quantitative normal cranial asymmetry in a population without pathology or functional disturbance is necessary to avoid malpractice. However, the work of Russo et al. [23] shows that an asymmetry index found in the skulls of all age groups showed no significant differences. When measures in millimeter are compared, no significant differences were detected between foetuses and newborns and also between two adult groups, meaning that close age groups have similar metric measures. Thus, the use of an index that supplies the data of asymmetry as percentage meaning quantitative difference among the sides is necessary. The position asymmetry of vessel and nerves foramina (carotid canal, foramina spinosum, ovale, and stylomastoid) verified in the whole sample and considered as normal corresponds to an average index of 4%, ranging from 2.6 to 6.6% [23].

Concerning the size of foramina, we have found numerous studies measuring the size of the jugular foramen as a function of the age, gender, and type of population studied. Skrzat et al. [24] reviewed the data collected by various studies observing this foramen in North American, Indian [25], Brazilian, and Nigerian populations. All these studies led to standard values being obtained for this foramen, along with the observation that the right foramen is larger than the left foramen in the great majority of cases. These values led Papini et al. [26] to compare them with the size of the jugular foramina of 53 patients with multiple sclerosis. The objective was to establish a link between internal jugular vein foraminal stenosis and the disease studied. The results demonstrate an average reduction of 10% in this foramen in patients affected. However, the authors do not observe any correlation between the severity of the lesions and the reduction in diameter of this foramen.

On the basis of these studies, our current clinical research is focusing on the observation of jugular foramen stenosis in patients with PCS and suffering from tension headaches. The foramen of the mid temporal fossa was studied by Sepahdari et al. [27]. They determined the first normative values for the temporal bone on the basis of 50 CT scans without facial or trigeminal nerve pathology. This research analyses and compares the size and the symmetry of the facial nerve canal, oval foramen, pterygoid canal, and foramen rotundum. It shows the importance of the notion of the foramen and canal symmetry for the diagnosis of tumour diseases, which are reflected by a significant increase in their diameter. The study recalls the clinical importance of the petrous part of the temporal bone, since it is in contact with cranial nerves V, VII, VIII, IX, X, and XI, the internal carotid artery and the jugular vein, to quote only the main elements. In addition, the petrous portion contains the semicircular canals, which positional symmetry plays a major role in balance. Close to these elements, the Eustachian tube has an important role in the regulation of intra- and extra-tympanic pressures in the auditory canal. Dizziness and tinnitus are frequently cited symptoms of PCS, encouraging us to examine the symmetry of the temporal petrous bone more closely.

Recently, Edwards et al. [28, 29] published two reviews of the anatomy and pathology of the skull base. These studies measured the amount of space available in each foramen crossed by a cranial nerve. The objective was to determine standard normative values in order to facilitate the diagnosis of basilar diseases. For each of these orifices, the authors identified the neurological clinical signs which can suggest a foramen pathologic size. A large proportion of these clinical signs are also symptoms of PCS.

### Case studies

**Fig. 2:**
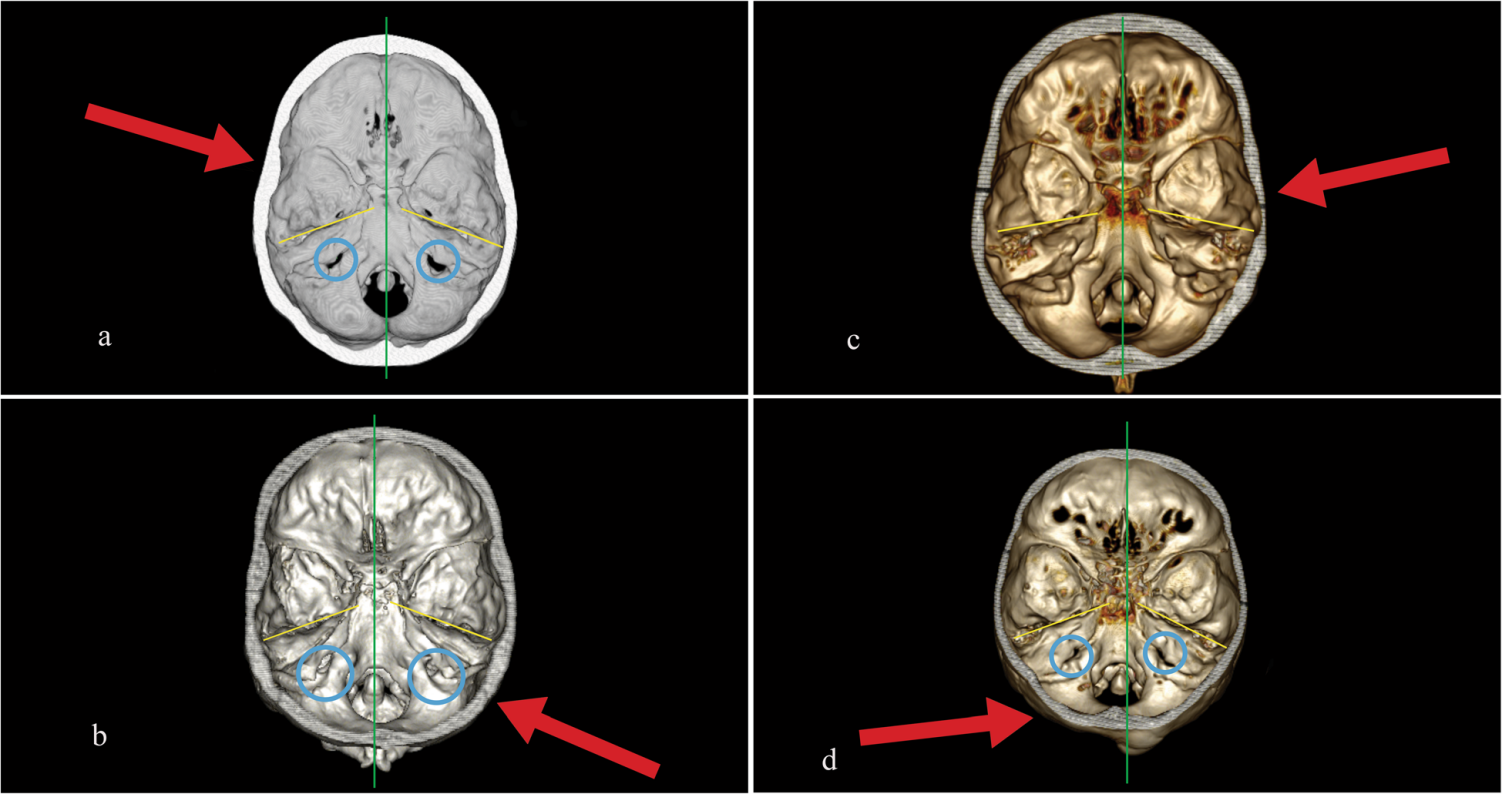
**a** Three-dimensional computed tomography (3D CT) of a 23-year-old woman 1 year after head trauma on the left temporal bone. Symptoms: tension-type headache on the left side, dizziness, chronic undernourishment. **b** 3D CT of a 43-year-old man 10 years after head trauma on the right temporo-occipital suture. Symptoms: severe tension-type headache on the right side, cervical pain. **c** 3D CT of a 41-year-old woman 1 month after head trauma on the temporal bone. Symptoms: dizziness. **d** 3D CT of a 47-year-old woman 3 years after trauma on the occipital bone. Symptoms: severe tension-type headache, dizziness, tinnitus. The red arrow shows the direction and the localisation of head impact. The green vertical axis allows us to see the asymmetry of the skull base. The blue rings show the size of the jugular foramen. The yellow lines appreciate the differences in the position of the petrous temporal bone

All four patients (Fig. 2a–d) were suffering from PCS with tension-type headache or dizziness. In Table 3, the left columns report the measures of the size of both jugular foramina. For better comprehension, we have noted the jugular foramen which is on the trauma side and on the healthy side. An asymmetry index larger than 20% was observed when patients suffering a tension-type headache. Even if the right foramen was frequently bigger than the left foramen, in case b, it was smaller than 26%. In case c, the variation between both foramina is 8%, the lowest value of this column and the patient had no tension-type headache.

**Table 3:** Measures of asymmetry skull base

Measures	Jugular foramen perimeter (mm)			Angle of the temporal bone		
	Trauma side	Healthy side	Trauma side/healthy side	Trauma side	Healthy side	Healthy side/trauma side
Case a	27.9	37.2	0.75 (25%)	28°	21°	0.75 (25%)
Case b	27.5	36.9	0.74 (26%)	21°	20°	0.95 (5%)
Case c	27.4	29.5	0.92 (8%)	37,7°	31,7	0.84 (16%)
Case d	29.11	36.3	0.8 (20%)	21,2	25,6	1.2 (20%)

On the right column, we reported the angle between both the temporal petrous bones and the middle axis of the skull base. An asymmetry index larger than 16% was observed when patients suffering dizziness (in case b, the variation was only 5% and dizziness was not reported).

## Discussion

Studies relative to measurements of the skull resistance demonstrate that the skull is a heterogeneous structure with anisotropic resistance. These results make it possible to optimise the modelling of the skull structure reactions to a trauma. The base of the skull is a region of the major bone and sutures shuffle and can be deformed following a head injury, particularly in children, as shown by statistical studies. This part of the skull is composed of numerous foramina, crossed by many neurovascular elements that may be directly or indirectly related to some of the symptoms of PCS.

The structure of the skull sustains significant impact as a result of kinetic energy transfer during a cranial trauma, to protect the integrity of the brain tissue. The neurovascular and neuroimaging studies may explain the interface between the bones, nerves and the brain in MTBI. Coello et al. [30] discussed the cranial nerve injury after minor head trauma. They used 2D CT and concluded that trivial head trauma that causes a minor head injury can result in cranial nerve palsies with a similar distribution to moderate or severe head injuries [30]. The assessment of 3D CT after head trauma may explain more deformation that causes neurovascular injuries. Despite numerous studies concerning PCS, the exact radiological, anatomical or clinical entities involved have not yet to be thoroughly elucidated. This deficiency certainly remains a major obstacle when it comes to medicolegal recognition.

The main limitation of this review is that it cannot be considered exhaustive. It may not include some relevant studies. While the selection of studies and the way in which they were analysed may be considered subjective, it remains the result of many years of experience in clinical practice.

Our case studies allowed us to show examples of the role of anatomical skull variations influencing the effect of local impact and the PCS symptoms. In the absence of symptoms, we noted small differences. Differences are over 16% for petrous bone when there is dizziness, over 20% for the jugular foramen in the presence of tension-type headaches. We are aware that our opinion needs more data to be proven with statistical significance on a large-scale base. These measures can vary depending on the angle of view. Illustrations are only in 2D, even if the picture was extracted and converted by 3D analysis. For a better biomechanical comprehension, only an analysis in 3D with different points of view allows a real understanding of how the skull may be deformed by a trauma occurring in three dimensions by definition. These values may confirm the biomechanical link between skull trauma and tension-type headaches or dizziness. At present, the first-case studies performed are providing new data to help us understand the symptoms described by our patients better.

The analysis presented in this article brought together numerous elements supporting our stated hypothesis. Future studies will probably enable us to provide additional understanding when it comes to related current medicolegal debates and issues.

## Data Availability

All data analysed during this study are included in this article.

## Abbreviations

2D: Two-dimensional
3D: Three-dimensional
CT: Computed tomography
FEM: Finite element model
MTBI: Mild traumatic brain injuries
PCS: Post-concussion syndrome
TBI: Traumatic brain injuries
